# Heat Shock Protein 70 Inhibits the Activity of Influenza A Virus Ribonucleoprotein and Blocks the Replication of Virus *In Vitro* and *In Vivo*

**DOI:** 10.1371/journal.pone.0016546

**Published:** 2011-02-24

**Authors:** Gang Li, Junjie Zhang, Xiaomei Tong, Wenjun Liu, Xin Ye

**Affiliations:** 1 Center for Molecular Immunology, CAS Key Laboratory of Pathogenic Microbiology and Immunology, Institute of Microbiology, Chinese Academy of Sciences (CAS), Beijing, P. R. China; 2 Center for Molecular Virology, CAS Key Laboratory of Pathogenic Microbiology and Immunology, Institute of Microbiology, Chinese Academy of Sciences (CAS), Beijing, P. R. China; 3 Graduate University of Chinese Academy of Sciences, Beijing, P. R. China; Institut Pasteur, France

## Abstract

**Background:**

Heat shock protein 70 (Hsp70) was identified as a cellular interaction partner of the influenza virus ribonucleoprotein (RNP) complex. The biological significance of the interaction between Hsp70 and RNP has not been fully investigated.

**Principal Findings:**

Here we demonstrated that Hsp70 was involved in the regulation of influenza A viral transcription and replication. It was found that Hsp70 was associated with viral RNP by directly interacting with the PB1 and PB2 subunits, and the ATPase domain of Hsp70 was required for the association. Immunofluorescence analysis showed that Hsp70 was translocated from the cytoplasm into the nucleus in infected cells. Then we found that Hsp70 negatively regulated the expression of viral proteins in infected cells. Real-time PCR analysis revealed that the transcription and replication of all eight viral segments were significantly reduced in Hsp70 overexpressed cells and greatly increased as Hsp70 was knocked down by RNA interference. Luciferase assay showed that overexpression of Hsp70 could inhibit the viral RNP activity on both vRNA and cRNA promoters. Biochemical analysis demonstrated that Hsp70 interfered with the integrity of RNP. Furthermore, delivered Hsp70 could inhibit the replication of influenza A virus in mice.

**Significance:**

Our study indicated that Hsp70 interacted with PB1 and PB2 of RNP and could interfere with the integrity of RNP and block the virus replication *in vitro* and *in vivo* possibly through disrupting the binding of viral polymerase with viral RNA.

## Introduction

Influenza A virus is a single-stranded, negative-sense RNA virus with eight genomes, belonging to the family *Orthomyxoviridae*. In the nucleus, eight genomes are capsidated by multiple copies of nucleoproteins and viral heterotrimeric polymerase complex, including PA, PB1, and PB2. The complex, namely viral RNP, is required for viral transcription and replication. The transcription of vRNA is initiated by use of short capped RNA oligonucleotides as primers which are snatched from host cellular mRNA by viral polymerase. Such primers contain the cap structure at the 5′ ends followed by 9–15 nucleotides [1]. The transcription terminates at a sequence of five to seven uridines located 15 to 17 nucleotides from the 5′ end of vRNA templates, where viral polymerase complex stutters by the addition of poly(A) tail. In contrast, the replication includes the intermediate complementary RNA synthesis which is positive-sense RNA and serves as the template for synthesis of progeny vRNA. The replication does not need primer and guarantees the fidelity.

Though the transcription and replication are fulfilled by viral polymerase complex, the three subunits of RNP have different functions. PB1 subunit is the core of the complex and has the polymerase activity [2], [3], and vRNA and cRNA promoter binding activities [4], [5], while PB2 is responsible for cap recognition [6], [7]. PA has multiple functions such as protease activity causing the degradation of its associated partner and itself [8], endonuclease activity [9], [10], promoter binding activity [11], [12], virus assembly [13], and N-terminal of PA is involved in cap binding [14].

Some host factors are involved in replication and transcription of the influenza virus genome [15]. RanBP5 was found to interact with either PB1 or PA-PB1 heterodimer and help PA-PB1 dimer to enter the nucleus to form PA-PB1-PB2 complex [16]. Ebp1 was identified to interact with PB1 subunit and interfered with viral RNA synthesis in vitro, indicating that it was an inhibitor of influenza virus polymerase [17]. In addition, Hsp90 was identified to stimulate viral RNA polymerase activity by interacting with PB2 or PB1-PB2 dimer and promoting them to enter the nucleus and form the functional polymerase complex [18], [19]. Additionally, host factor CCT was identified as PB2 binding protein, and the silencing of CCT reduced viral replication by reducing PB2 protein level and RNA accumulation [20].

Hsp70 is a molecular chaperone belonging to heat shock protein family. It is a stress-inducible member, in contrast to Hsc70 expressed constitutively. Hsp70 plays important role in the folding and assembly of newly synthesized proteins, refolding of misfolded and aggregated proteins, membrane translocation of organellar and secretory proteins, and controlling of the activities of regulatory proteins [21], [22], [23], [24]. Hsp70 consists of a N-terminal ATPase domain and a C-terminal peptide binding domain (PBD) in which there is EEVD motif enabling it to bind to co-chaperones and other heat shock proteins such as Hsp90, Hsp40 and Hop [25], [26], [27], [28]. Hsp70 was found to inhibit virus infection [29], [30], [31]. Nevertheless, some viruses could utilize Hsp70 to help their own replication [32], [33], [34], [35], [36], [37]. For influenza virus Hsp70 was identified to be involved in regulation of viral life cycle. Hsc70 interacted with M1 and the interaction was required for viral production [38]. Hsp70 was found to bind to ribonucleoprotein complex, which prevented M1 from binding to vRNP so that the nuclear export of vRNP was inhibited [39]. Proteomic data confirmed that Hsp70 was the interaction partner of influenza virus ribonucleoprotein complex [40]. It was reported that a higher level of Hsp70 was associated with vRNPs of influenza A/Ann Arbor/6/60 virus which was defective in replication compared with those of rWT virus [41]. However, it is not clear whether the interaction between Hsp70 and RNP has any effect on the polymerase activity.

In this study, we demonstrated that Hsp70 negatively regulated influenza virus RNP activity. It interacted with PB1 and PB2 subunits of RNP and was translocated from the cytoplasm to the nucleus in infected cells. Furthermore, Hsp70 interfered with the integrity of RNP, and then negatively regulated the viral transcription and replication.

## Results

### Hsp70 interacted with PB1 and PB2 subunits of RNP

**Figure 1:**
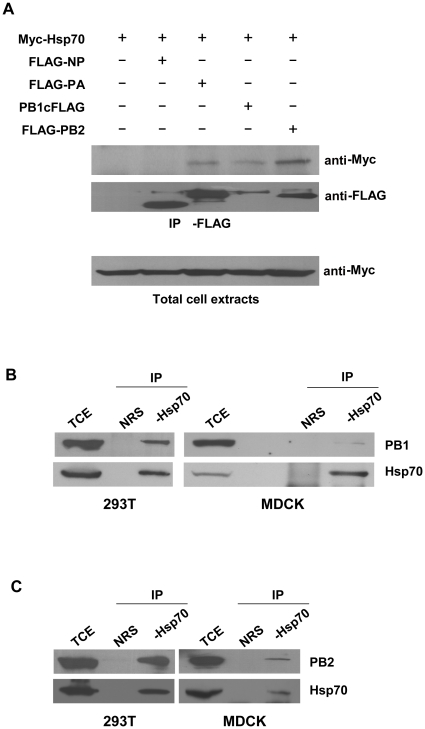
Hsp70 interacts with PB1 and PB2 subunits of viral polymerase complex. (A) 293T cells were transfected with pCMV-Myc-Hsp70 and pcDNA3-FLAG-NP, pcDNA3-FLAG-PA, pcDNA3-PB1cFLAG, or pcDNA3-FLAG-PB2, with pcDNA3.1 as negative control. The total cell extracts were immunoprecipitated with anti-FLAG antibody and immunoblotted with monoclonal anti-Myc antibody. (B, C) 293T cells and MDCK cells were infected with rWSN at MOI of 1 for 24 h. Total cell extracts were immunoprecipitated with rabbit anti-Hsp70 antibody or normal rabbit serum (NRS) as control and immunoblotted with goat anti-PB1 antibody (B) or goat anti-PB2 antibody (C). These experiments were repeated twice with identical results.

It was known that Hsp70 was associated with RNP complex [39], [40]. To understand the significance of the association, we examined which subunit(s) of RNP that Hsp70 interacts with. 293T cells were co-transfected by Myc-Hsp70 with FLAG-NP, FLAG-PA, PB1cFLAG, or FLAG-PB2 plasmid, respectively. The total cell extracts were prepared and immunoprecipitated with anti-FLAG antibody followed by immunoblotting with anti-Myc antibody. The result showed that Hsp70 interacted with PA, PB1, and PB2 but not NP (Fig. 1A). We also examined whether endogenous Hsp70 could interact with PA, PB1, and PB2 in infected cells. 293T and MDCK cells were infected, and then the total cell extracts were prepared and subjected to immunoprecipitation with anti-Hsp70 antibody and immunoblotting with anti-PA, anti-PB1 or anti-PB2 antibody. The results showed that both PB1 and PB2 (Fig. 1B, C) interacted with endogenous Hsp70 in both cell lines. There was no detectable interaction between PA and endogenous Hsp70 (data not shown).

**Figure 2:**
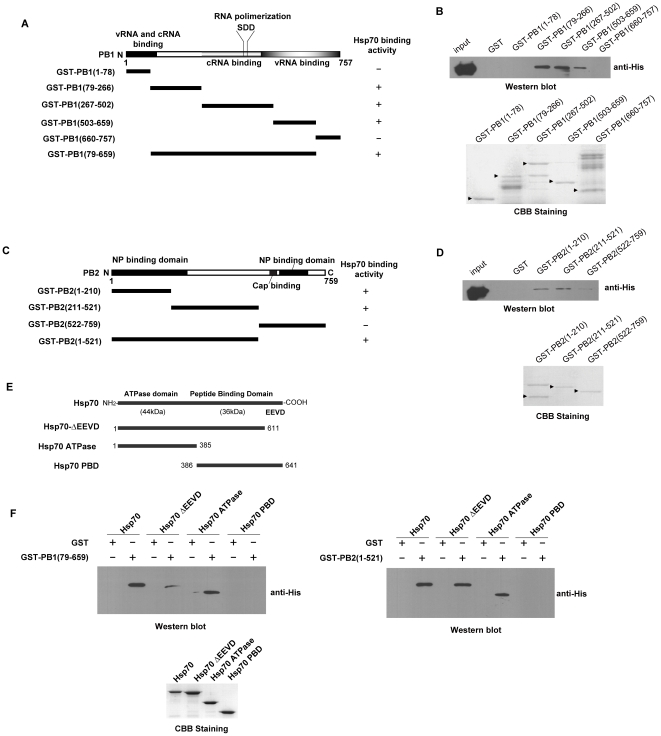
Hsp70 interacts with PB1 and PB2 directly. The GST-fused PB1 mutants (A, B) or GST-fused PB2 (C, D) mutants were generated. The interaction between GST-PB1 mutants (B) or GST-PB2 mutants (D) and His-Hsp70 was analysed by GST pull-down assay and Western blot (upper panel), the CBB staining of purified proteins were shown in lower panel. (A, C) Binding activity is indicated as “+”, no significant binding activity as “−”. (E) The schematic map of Hsp70 and its mutants. (F) His-Hsp70, His-Hsp70ΔEEVD, His-Hsp70 ATPase domain and His-Hsp70 PBD were prepared and subjected to GST pull-down assays with GST-PB1(79–659) (upper left panel), GST-PB2(1–521) (upper right panel) and GST as negative control, and the purified His-Hsp70 and its mutants were subjected to CBB staining (lower panel). Arrows indicated purified proteins of CBB staining. These experiments were repeated twice with identical results.

GST pull-down assays were carried out to confirm the direct interaction of Hsp70 with PB1 and PB2. As shown in Fig. 2A, B Hsp70 interacted with PB1 (aa79-659) which included cRNA promoter binding domain, part of vRNA promoter binding domain, and RNA polimerization motif [5], [42]. The data in Fig. 2C, D illustrated that Hsp70 interacted with N-terminal domain (aa1-521) of PB2 which included the NP binding domain [43].

Hsp70 could be divided into two functional domains, the N-terminal ATPase domain and the C-terminal protein binding domain (PBD) in which there was EEDV motif (Fig. 2E). To examine which domain of Hsp70 was required for its interaction with PB1 and PB2, full length and truncated forms of Hsp70 were generated for GST pull-down assay. As shown in Fig. 2F, the ATPase domain was required for its direct interaction with PB1 (aa79-659) and PB2 (aa1-521).

### Hsp70 was translocated into nucleus post-infection

**Figure 3:**
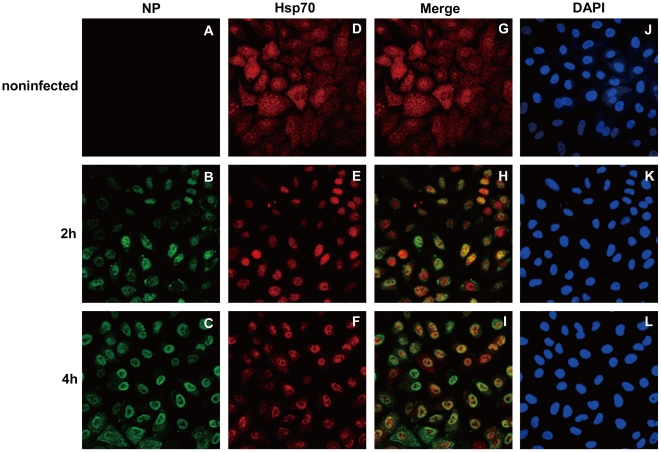
Hsp70 is translocated into the nucleus in infected A549 cells. A549 cells were infected with rWSN at MOI of 5 for 1 h and cultured for indicated time. The infected and noninfected A549 cells were fixed and stained with rat anti-NP antibody (A, B and C) and rabbit anti-Hsp70 antibody (D, E and F), colocalization of Hsp70 and NP were shown in merged images (G, H and I), and DAPI staining indicated the nucleus (J, K and L).

The subcellular localization of Hsp70 during infection was examined by immunofluorescence assay with A549 cells. Endogenous Hsp70 was mainly localized in the cytoplasm of non-infected cells (Fig. 3D). In contrast, Hsp70 was translocated into the nucleus at 2 h post-infection (Fig. 3E). The staining of NP indicated the nuclear localization of viral polymerase complex (Fig. 3B). NP was localized in the nucleus of most of cells at 4 h post-infection (Fig. 3C), and colocalized with Hsp70 in the nucleus (Fig. 3H, I). These results demonstrated that Hsp70 was translocated into the nucleus and colocalized with viral polymerase where the viral transcription and replication occurred. The same result could also be reproduced with HeLa cells (data not shown).

### Induction of Hsp70 with PGA1 inhibited the transcription and replication of influenza A virus genomes

**Figure 4:**
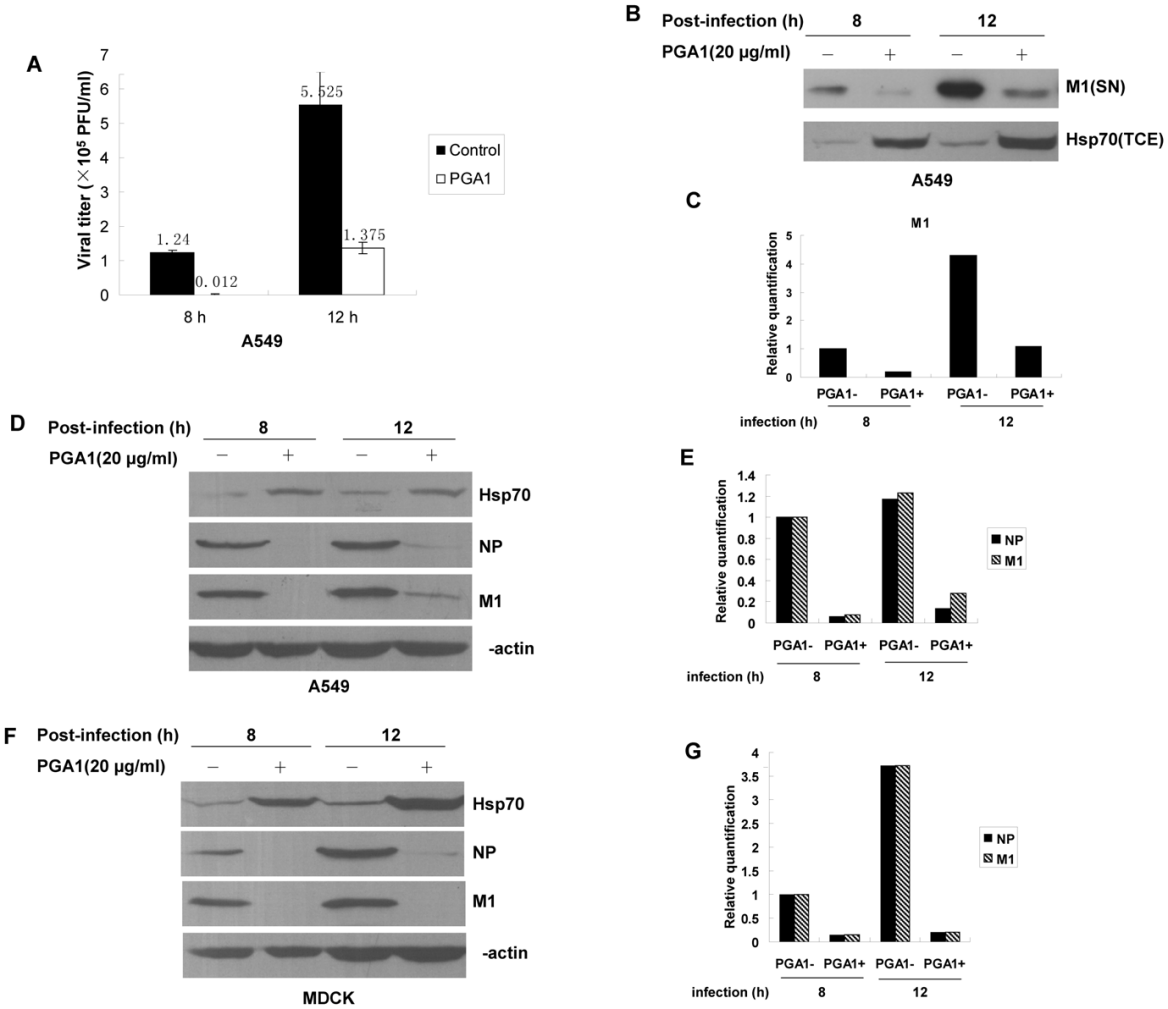
PGA1 treatment inhibits the propagation of influenza A virus. Cells were infected with rWSN at MOI of 1, then cultured in the absence or presence of PGA1 for the indicated intervals. The supernatants and total cell extracts were harvested. (A) The supernatants from infected A549 cells were subjected to plaque assay to measure the viral titers shown as the data on the bars. Error bars represent standard deviation (n = 3). (B, C) M1 protein (SN) and Hsp70 (TCE) were detected by immunoblotting (B) with mouse anti-M1 and rabbit anti-Hsp70 antibodies (SN and TCE stand for supernatant and total cell extract, respectively). (C) The relative quantification of M1 in (B). (D–G) Total cell extracts of infected A549 cells (D, E) and MDCK cells (F, G) were prepared for immunoblotting with rabbit anti-Hsp70, rat anti-NP, mouse anti-M1, and rabbit anti-β-actin antibodies (D, F). (E, G) The relative quantification of M1 and NP in (D, F).

In order to understand the biological function of Hsp70 on influenza virus replication, cells were infected, treated with PGA1, which was known to induce the overexpression of Hsp70 as reported [44]. The supernatants were harvested to examine the viral titers by plaque assay at different time points post-infection. The data showed that the viral titers in the supernatants from PGA1-treated A549 cells were reduced at 8 h and 12 h post-infection in comparison with the control (Fig. 4A), which was consistent with the result of others [39]. The reduced M1 protein level from the virion of the supernatants confirmed that the PGA1-treatment caused the decrease of released virion (Fig. 4B, C).

We wondered if the effect of Hsp70 on the viral titers was due to the decreased level of viral proteins. The total cell extracts were prepared at indicated time points for immunoblotting with anti-Hsp70, anti-M1, anti-NP, and anti-β-actin antibodies. The protein level of Hsp70 increased as expected while both M1 and NP protein levels were reduced in PGA1-treated A549 (Fig. 4D, E) and MDCK (Fig. 4F, G) cells in comparison with those in untreated cells.

**Figure 5:**
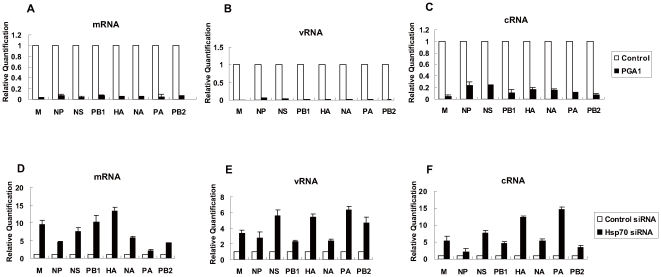
Hsp70 negatively regulates influenza virus RNA transcription and replication. (A, B, C) A549 cells were infected with rWSN at MOI of 1, and then cultured in the absence or presence of PGA1 for 8 h. The cells were harvested and the total RNAs were isolated with TRIzol reagent, then mRNA (A), vRNA (B) and cRNA (C) of eight genomes were quantified by real-time RT-PCR as described in Materials and Methods. The white bars of nontreated samples are set to a relative value of 1, and the black bars are expressed as relative value compared with control. (D, E, F) HeLa cells were transfected with Hsp70 siRNA or control siRNA for 60 h and infected with rWSN at MOI of 1 for 7 h. The total RNAs were isolated and quantified by real-time RT-PCR for viral mRNA (D), vRNA (E) and cRNA (F) of eight genomes. The white bars of control siRNA-transfected cells are set to a relative value of 1, and the black bars represent the relative value compared with control. The data represent the means of three independent experiments, each performed in triplicate. Error bars represent standard deviation (n = 3).

To determine whether the inhibitory effect of PGA1 on viral proteins level was due to the inhibition of the transcription and replication of viral genomes, the amounts of mRNA, vRNA and cRNA of eight genomes were measured. A549 cells were infected and then treated with PGA1. The total RNAs were prepared at 8 h post-infection and the amounts of mRNA, vRNA, and cRNA were quantified by real-time RT-PCR. The data showed that mRNA, vRNA, and cRNA levels of all eight genomes were greatly reduced in PGA1-treated cells (Fig. 5A, B, C). These results indicated that PGA1 treatment inhibited viral transcription and replication so as to reduce viral proteins level and viral titer.

### The transcription and replication of influenza A virus genomes were up-regulated in Hsp70 depleted cells

**Figure 6:**
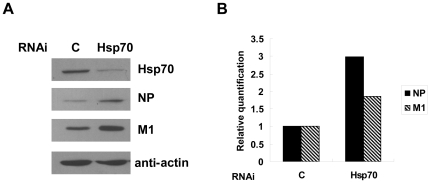
The influenza virus proteins level is increased in Hsp70 depleted cells. HeLa cells were transfected with Hsp70 siRNA or control siRNA and infected with rWSN for 7 h. Total cell extracts were prepared for immunoblotting with indicated antibodies (A), and the amount of proteins were quantified (B).

To confirm that the inhibitory effect of PGA1 on the viral transcription and replication is indeed due to the induction of Hsp70, we took the approach of RNA interference to knock down the endogenous Hsp70 and analyze the viral transcription and replication. HeLa cells were used in this experiment because the siRNA can efficiently knock down the expression of Hsp70 in this cell line. HeLa cells were transfected with Hsp70 siRNA or control siRNA, and then infected with influenza A virus for 7 h. Total RNAs were isolated and subjected to real-time RT-PCR for mRNA, vRNA and cRNA. The results showed that the mRNA, vRNA, and cRNA levels of all eight genomes increased in Hsp70 depleted cells compared with those in control cells (Fig. 5D, E, F). Consistently the protein levels of M1 and NP also increased in Hsp70-depleted cells (Fig. 6A, B). These results suggested that knockdown of Hsp70 favored viral transcription and replication.

### Hsp70 inhibited both transcription and replication of RNP

**Figure 7:**
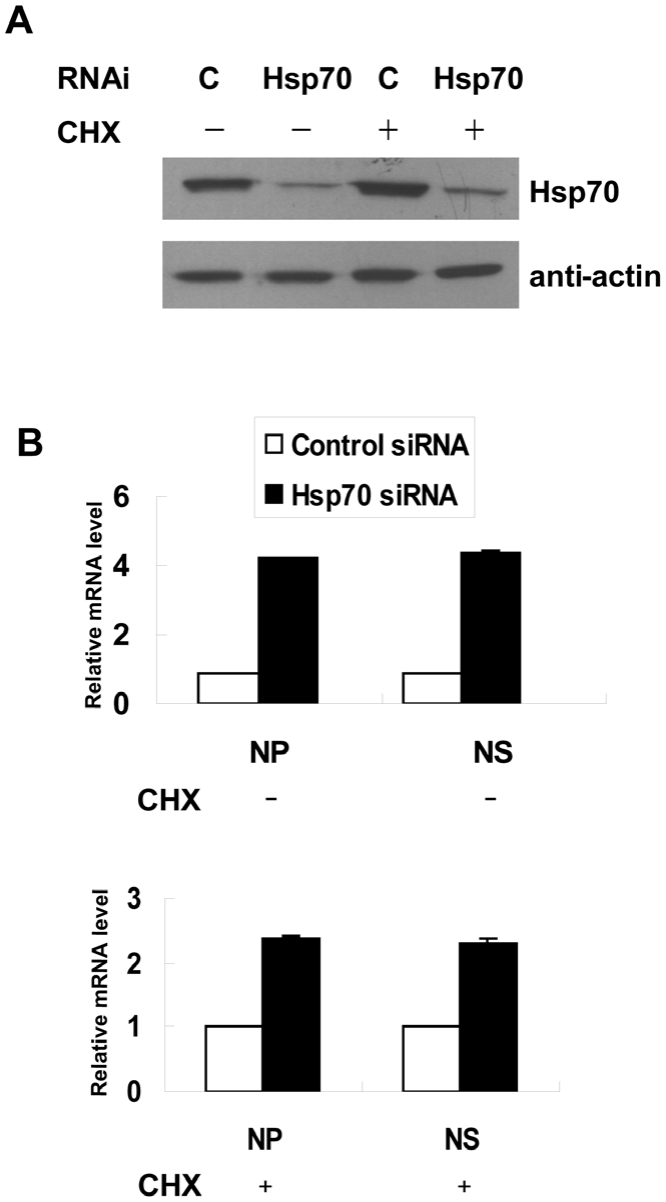
Hsp70 inhibits both transcription and replication of RNPs. HeLa cells were transfected with Hsp70 siRNA and control siRNA for 60 h and infected with rWSN in the presence of 100 µg/ml of CHX for 1 h then cultured with DMEM containing CHX for 7 h, or in the absence of CHX as control. Total cell lysates were prepared for immunoblotting with anti-Hsp70 and anti-β-actin antibodies (A). Total RNA was isolated and subjected to real-time RT-PCR to measure the amounts of mRNA of NP and NS segments. The white bars of control siRNA-transfected cells are set to a relative value of 1, and the black bars represent the relative value campared with control (B). The data represent the means of three independent experiments, each performed in triplicate. Error bars represent standard deviation (n = 3).

The primary transcription of input vRNP initiates when host cell is infected with influenza virus, followed by the replication producing progeny vRNP which is used to carry on subsequent secondary transcription. Since mRNA, vRNA, and cRNA levels increased in Hsp70-depleted HeLa cells, we wondered whether the increased mRNA level was caused by increased progeny vRNA or the direct effect of Hsp70 on primary transcription of input vRNP. It has been reported that cycloheximide (CHX), an inhibitor of protein synthesis, inhibited the transition from primary transcription to replication [45], therefore we could examine whether Hsp70 affect the primary transcription by using CHX to block the synthesis of progeny vRNP. CHX treatment did not affect the knockdown of endogenous Hsp70 in HeLa cells (Fig. 7A), then real-time RT-PCR results showed that the amounts of mRNA of NP and NS segments increased 2–3 fold in the presence of CHX and 4–5 fold in the absence of CHX in Hsp70 depleted HeLa cells (Fig. 7B). These data indicated that removing Hsp70 in the absence of replication caused the increase of mRNA level, but the increase was higher if both transcription and replication occured, suggesting that Hsp70 negatively regulated both transcription and replication.

### Hsp70 inhibited the activity of the influenza virus polymerase in a dose-dependent manner

**Figure 8:**
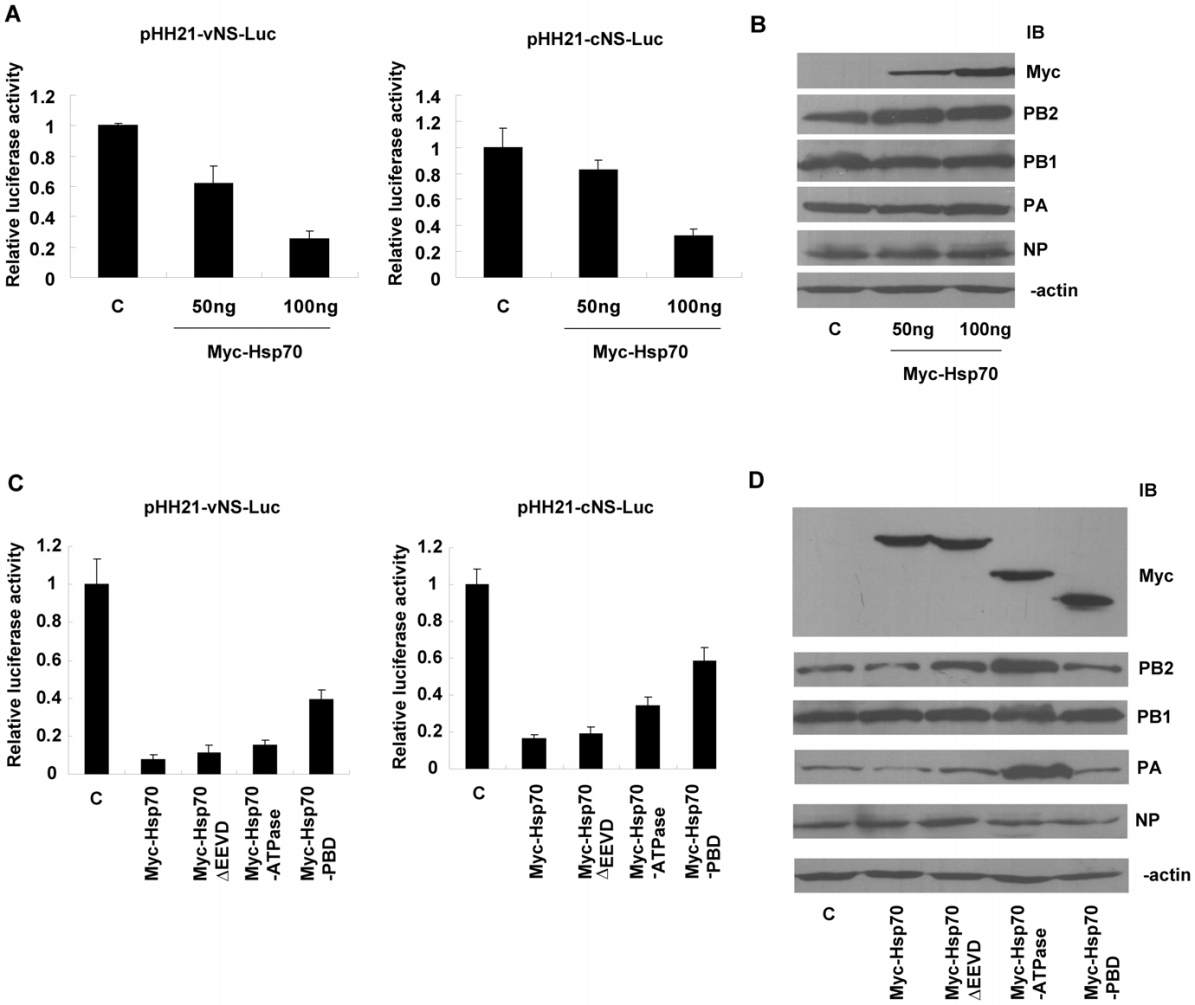
Hsp70 inhibits influenza virus polymerase activity in a dose-dependent manner. (A, B) 293T cells were transfected with pHH21-vNS-Luc (left panel) or pHH21-cNS-Luc (right panel), NP, PA, PB1, PB2 expression plasmids, and 50 ng, 100 ng, 200 ng of pCMV-Myc-Hsp70 or 200 ng of pcDNA3.1 as negative control, then subjected to luciferase assay (A) and immunoblotting with indicated antibodies (B). (C) 293T cells were transfected with pHH21-vNS-Luc (left panel) or pHH21-cNS-Luc (right panel), plasmids encoding NP, PA, PB1, PB2, and 200 ng of pCMV-Myc-Hsp70, pCMV-Myc-Hsp70ΔEEVD, pCMV-Myc-Hsp70 ATPase domain, pCMV-Myc-Hsp70 PBD or 200 ng of pcDNA3.1 as negative control. The luciferase assays were performed accordingly. The data represent the means of two independent experiments, each performed in triplicate. Error bars represent standard deviation (n = 3). (D) The protein levels were analysed by immunoblotting with indicated antibodies.

To examine whether Hsp70 could regulate the activity of influenza virus polymerase, we took the approach of luciferase assay with vRNA and cRNA promoters. 293T cells were co-transfected with multiple plasmids as followed (i) the expression vectors encoding influenza virus polymerase subunits and NP; (ii) pHH21-vNS-Luc or pHH21-cNS-Luc reporter plasmid; and (iii) pCMV-Myc-Hsp70 in different doses or pcDNA3.1 as negative control. The cell lysates were subjected to luciferase assay and Western blot. The data of luciferase assay showed that relative luciferase activities under both vRNA and cRNA promoters in Hsp70 overexpressed cells were much lower than those in control cells in a dose-dependent manner (Fig. 8A), which indicated that Hsp70 inhibited the activity of viral polymerase complex on both vRNA and cRNA promoters. The immunoblotting results showed that the levels of RNP proteins were similar in the cells transfected with or without Myc-Hsp70 (Fig. 8B).

To examine which domain of Hsp70 was required for the inhibitory effect, we generated the truncated forms of Hsp70 and performed luciferase assay. The data showed that ATPase domain had similar inhibitory activity as full length, PBD partially inhibited the activity of viral polymerase but EEVD motif was not required (Fig. 8C). The immunoblotting data showed that the expression of RNP subunits and truncated forms of Hsp70 in transfected cells were similar except that the expressions of PA and PB2 were higher in Myc-Hsp70 ATPase-transfected cells (Fig. 8D).

### Hsp70 interfered with the integrity of RNP

**Figure 9:**
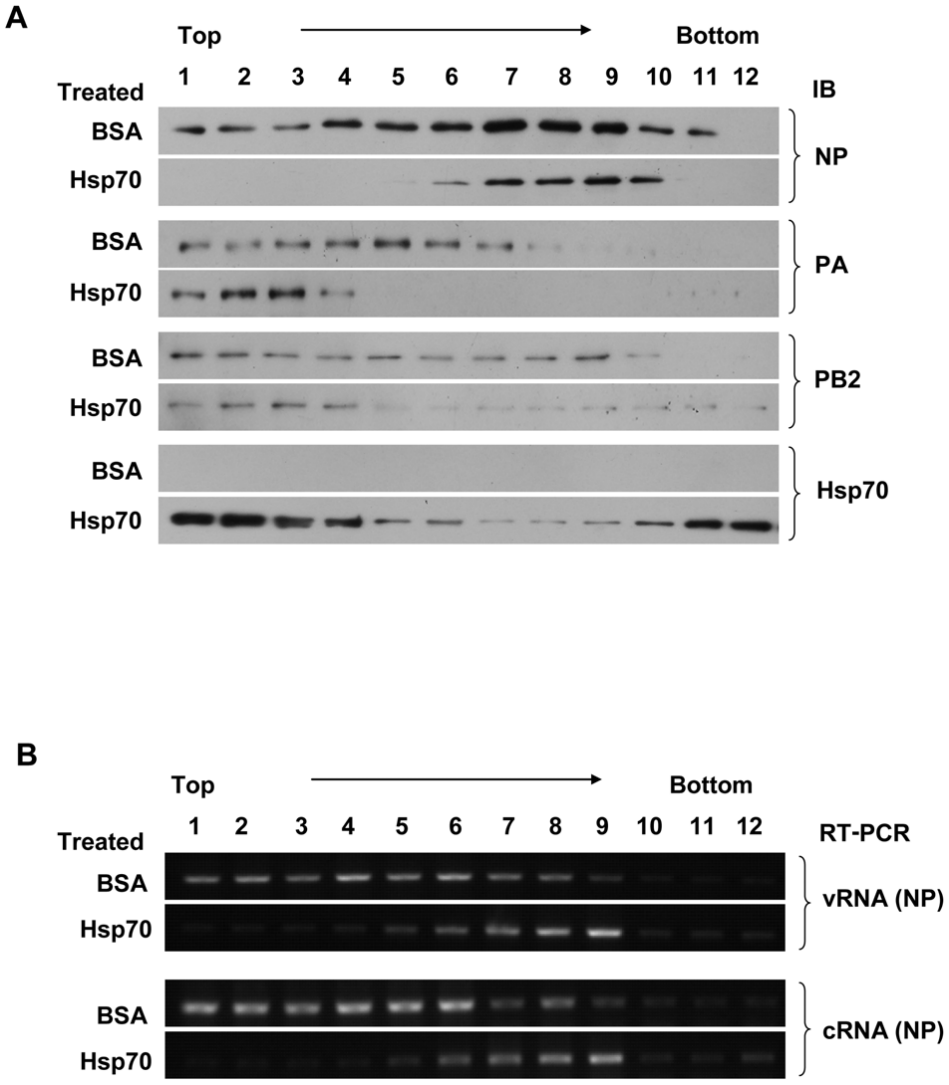
Hsp70 interferes with the integrity of RNP. (A, B) MDCK cells were infected with rWSN, and then subjected to cell fractionation. The nuclear fraction was incubated with His-Hsp70 or with BSA as control, and then applied to a glycerol gradient. Fractions (1 to 12) were collected from the top to the bottom. An aliquot of each fraction was subjected to immunoblotting with indicated antibodies (A). RNA was purified from each fraction, and reverse transcribed into cDNA with primer specific for vRNA or cRNA. The cDNA was then subjected to semiquantitative PCR with primers for NP. The PCR products were subjected to 1% agarose gel and visualized by ethidium bromide (B). These experiments were repeated three times with identical results.

The viral polymerase complex consists of PA, PB1 and PB2, in which PB1 and PB2 interacted with Hsp70 (Fig. 1). Furthermore the binding regions of PB1 and PB2 with Hsp70 (Fig. 2) were pivotal to the RNP functions. We speculated that Hsp70 might affect the integrity of RNP. To test this idea, the MDCK cells were infected with virus and the nuclear fraction was isolated and incubated with His-Hsp70 or BSA as negative control. Then the mixtures were subjected to glycerol gradient centrifugation. The fractions were collected and subjected to immunoblotting with anti-NP, anti-PA, anti-PB2 and anti-Hsp70 antibodies (Fig. 9A) as well as semiquantitative RT-PCR for vRNA and cRNA of NP segment (Fig. 9B). As shown in Fig. 9A, in BSA-treated RNPs PA sedimented predominantly in fractions 1 to 7, PB2 in fractions 1 to 9, NP in almost all fractions except fraction 12, then in Fig. 9B vRNA and cRNA sedimented in fractions 1 to 8, therefore, it was shown that RNP complex sedimented to fractions 1 to 7, because there existed vRNA (cRNA), NP, PA and PB2. In contrast, in Hsp70-treated RNPs vRNA and cRNA sedimented mainly in fractions 6 to 9, NP in fractions 6 to 10, but PA and PB2 in fractions 1 to 4 where Hsp70 sedimented. We concluded that the addition of Hsp70 disrupted the integrity of RNP, with NP and vRNA (cRNA) binding together, but PA and PB2 were not in the same fractions.

### Hsp70 inhibited influenza A virus replication *in vivo*

**Figure 10:**
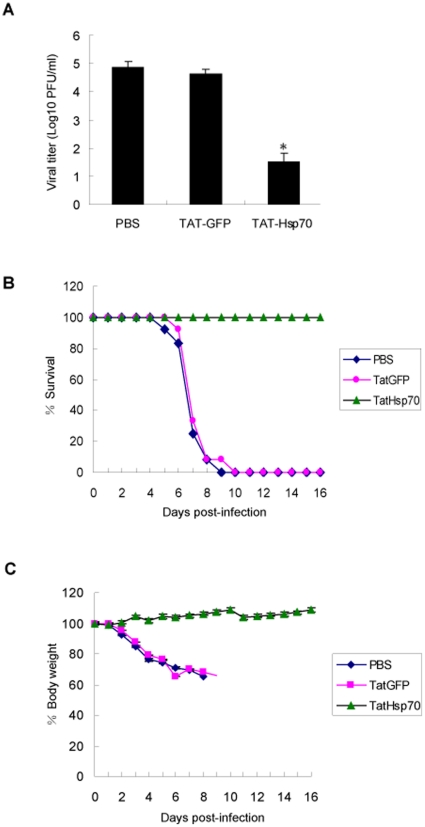
Hsp70 inhibits influenza virus replication in vivo. Four-week-old female BALB/c mice were infected rWSN plus PBS, TAT-GFP or TAT-Hsp70, and then injected intraperitoneally with PBS, TAT-GFP or TAT-Hsp70 daily from day 1 to 6 post-infection. Three mice from each group were sacrificed on day 3 post-infection, the viral titers in lungs were analysed by plaque assay (A). The survival (B) and body weight (C) of the remaining mice were monitored daily. Asterisk indicates a *P* value of<0.01 for viral titer comparing TAT-Hsp70 treated group to PBS and TAT-GFP treated groups. Experiments were performed twice with identical results. Error bars represent standard deviation (n = 3).

In order to investigate whether Hsp70 protein inhibits influenza virus replication in vivo, we prepared the TAT-Hsp70 protein for virus infection assay. It had been reported that TAT-Hsp70 was able to be delivered into cells and mice efficiently [46], [47]. We examined the effect of TAT-Hsp70 on viral infection in BALB/c mice. Preliminary experiments with mice infection showed that TAT-Hsp70 protein had not exerted any effect on the virus when they were mixed before intranasal infection (data not shown). On day 3 post-infection the viral lung titers of the TAT-Hsp70-treated mice were much lower than those of control mice treated with PBS or TAT-GFP, 7.21×10^4^, 4.12×10^4^ and 33 PFU/0.2 g lung in PBS, TAT-GFP and TAT-Hsp70-treated mice respectively. As shown in Fig. 10C, the body weights of control groups and TAT-Hsp70-treated group have diverged significantly on day 3 post-infection. The survival and body weight were examined daily post-infection. All of TAT-Hsp70-treated mice survived while all of PBS or TAT-GFP-treated mice died on the ninth or tenth day respectively (Fig. 10B). The body weight of TAT-Hsp70-treated group increased but those of control groups declined greatly (Fig. 10C). These results showed that Hsp70 protein could effectively inhibit virus propagation in mice and protect the mice from influenza virus infection.

## Discussion

In this study, we revealed a distinct mechanism of Hsp70-mediated regulation of influenza virus polymerase activity. Previous reports had suggested that Hsp70 could inhibit the viral propagation through blocking the nuclear export of ribonucleoprotein complex [39]. In light of these and our current findings, Hsp70 may have multiple mechanisms of controlling the viral replication during virus infection. Although more studies are needed to understand the detailed regulatory mechanisms of Hsp70 in viral replication, our findings revealed the novel role of Hsp70 in regulating viral polymerase activity by interfering with the integrity of RNP.

Co-immunoprecipitation assays showed that Hsp70 could interact with PA subunit of influenza virus polymerase in cells transfected with Hsp70 and PA (Fig. 1A) but the interaction between Hsp70 and PA was undetectable in infected 293T and MDCK cells. The reason may be that the interaction between PA and Hsp70 was relatively weak and the amount of PA protein was much lower in infected cells than that in transfected cells so it was hard to detect the interaction between them. Therefore further experiments such as GST pull-down assay were required to confirm their interaction.

The endogenous Hsp70 was located in cytoplasm [48]. In infected cells Hsp70 was translocated into nucleus where the viral transcription and replication occurred (Fig. 3). Therefore there was spatial possibility for Hsp70 to regulate the viral transcription and replication. But the exact mechanism of how Hsp70 was translocated into nucleus needs further elucidation.

Our results confirmed that Hsp70 interacted with PB1 and PB2 subunits both in vitro and in vivo (Fig. 1 and Fig. 2). We wondered how Hsp70 inhibited the function of RNP complex. Therefore we examined whether Hsp70 affected the integrity of RNP. The data in Fig. 9A, B showed that Hsp70 interfered with the binding of NP and vRNA (cRNA) with viral polymerase complex. In vitro GST pull-down assays affirmed that Hsp70 did not affect the binding of NP with PB1 and PB2 (data not shown). Furthermore, it was reported that PB1 bound to the vRNA and cRNA promoter [4], [5]. GST pull-down data (Fig. 2A) indicated that Hsp70 bound to vRNA and cRNA promoter binding domains of PB1. Therefore we speculated that Hsp70 disrupted the integrity of RNPs through interfering the binding of PB1 with viral RNA promoter so that viral RNA and RNA-bound NP fell off from the polymerase complex as shown in Fig. 9. Taken together, the results indicated that Hsp70 interfered with the integrity of RNP most likely by blocking the interaction of PB1 with vRNA (cRNA) rather than the association of NP with PB1 and PB2.

Inconsistently with our findings, Dalton RM reported that Hsp70 was not responsible for down-regulation of viral RNAs synthesis by using quercetin to inhibit Hsp70 synthesis [49], Quercetin has been proved to be involved in many cellular pathways in addition to the inhibition of Hsp70 synthesis. For example, quercetin inhibited NF-κB pathway [50]. It has been reported that NF-κB signaling regulated influenza virus RNA synthesis [51], so quercetin may inhibit viral RNAs synthesis by the inhibition of NF-κB pathway. Therefore, quercetin treatment in influenza virus infected cells may produce the counteractive effect on viral RNAs synthesis through Hsp70 and NF-κB pathway, or other unknown pathways, that may be the reason why Dalton, R. M. did not observe the effect of quercetin treatment on viral RNAs level.

Nowadays although there are some anti-influenza drugs available, but drug resistants emerged rapidly after therapeutic use in influenza virus infection [52]. It is necessary to develop new drugs against influenza virus. The peptide originated from viral protein as antiviral agent has been studied recently. It was reported that PB1_1–25_ peptide could inhibit the influenza virus polymerase activity [53], and the zinc finger peptide of M1(aa148–166) could block the propagation of influenza virus in mice [54]. Since the viral protein as a foreign protein may bring up side effect of immunoreactivity when being delivered in vivo, it will be better to use host factors as anti-influenza agents. It has been reported that Hsp70 induced by delta^12^-PGJ_2_ or PGA2 could inhibit influenza virus in vivo [55], [56]. It was known that prostaglandins could activate heat shock transcription factor which increased the transcription of both Hsp70 and Hsp90 [57]. But Hsp90 was proved to facilitate influenza virus propagation [18], [19]. Therefore prostaglandins were not ideal for anti-influenza drug. Recently TAT peptide was used to deliver exogenous protein into cultured cells and mice [58], [59], and it had no effect on influenza virus replication [60]. So we took the approach of TAT peptide mediated transduction method to deliver Hsp70 into mice and found that Hsp70 could inhibit the propagation of influenza virus in vivo. It was the first report that host protein was utilized as influenza virus inhibitor in vivo.

In conclusion, we demonstrate that Hsp70 is the inhibitory host factor for influenza virus by interfering with the viral polymerase activity. Our further research will be focused on the development of inhibitory peptide based on Hsp70 protein and providing the candidates for new anti-influenza drugs.

## Materials and Methods

### Plasmid construction

Hsp70 gene was amplified from human cDNA library by PCR and cloned into pCMV-Myc (Clonetech) and pET28c (Novagen). The truncated mutants of Hsp70 (ΔEEVD, aa1-611; ATPase domain, aa1-385; PBD, aa386-641) were inserted into pCMV-Myc and pET28c vectors. NP, PA and PB2 were cloned into pcDNA3-FLAG, and PB1cFLAG was inserted into pcDNA3.1 (Invitrogen) to generate expression vector for C-terminal FLAG-tagged PB1. The truncated mutants of PB1 and PB2 were cloned into pGEX-4T-2 (Amersham Pharmacia Biotech). pHH21-vNS-Luc was polymerase I expressing plasmid carrying an influenza virus-like RNA coding for the luciferase reporter protein (kindly provided by Martin Schwemmle, University of Freiburg) [53]. pHH21-cNS-Luc was generated by cloning cRNA promoter of NS with luciferase into pHH21.

### Cell lines, virus and antibodies

Madin-Darby Canine Kidney (MDCK) cells, human embryo kidney 293T cells, human lung adenocarcinoma epithelial cell line (A549) and HeLa cells were maintained at 37°C in Dulbecco's Modified Eagle's medium (DMEM, Gibco) supplemented with 10% heat-inactivated fetal bovine serum (FBS, PAA). The recombinant influenza virus A/WSN/33 (rWSN) was generated by 12 plasmids system as described previously [61]. Rabbit anti-Hsp70 and anti-PA antibodies were generated by immunizing the rabbits with GST-Hsp70 (aa618-633) and GST-PA (aa1-256), respectively, then affinity purified. Monoclonal anti-Myc (9E10) and anti-His antibodies, goat anti-PB1 (sc-17602), goat anti-PB2 (sc-17603) and rabbit anti-β-actin (sc-1616) antibodies were purchased from Santa Cruz Biotechnology. Mouse anti-FLAG (M2) antibody was purchased from Sigma. Mouse anti-M1 monoclonal antibody was prepared as described previously [62], Rat anti-NP antibody (generously provided by Wenjun Liu, Institute of Microbiology, Chinese Academy of Sciences) was generated by immunizing the rat with His-NP protein.

### Virus infection

Monolayer cultured cells were washed twice with PBS, and then infected with rWSN at an indicated multiplicity of infection (MOI). After virus adsorbtion for 1 h at 37°C, the cells were washed twice with PBS and incubated at 37°C with culture medium. As for PGA1 treatment, the cells were incubated with 20 µg/ml PGA1 (Sigma) in DMEM post-infection and harvested at indicated time.

### Plaque assay

MDCK monolayer cells in 35-mm dishes were washed with PBS, and serial dilutions of virus were adsorbed onto cells for 1 h. Unadsorbed virus was removed by washing with PBS, then monolayer cells were overlaid with DMEM supplemented with 3% low-melting-point agarose and 2 µg/ml TPCK-treated trypsin (Sigma). Visible plaques were counted and viral titers were calculated after 3 days of incubation. All data were expressed as the mean of triplicated samples.

### Transient transfection

293T cells were grown in 60-mm dishes at 80% confluence and transfected with plasmids using Lipofectamine 2000 transfection reagent (Invitrogen). The medium was replaced with fresh culture medium at 7 h post-transfection.

### Luciferase assay

293T cells were transiently transfected with plasmids expressing influenza virus NP, PA, PB1, PB2 (100 ng for each) and pHH21-vNS-Luc or pHH21-cNS-Luc (50 ng) with pCMV-β-gal plasmid (50 ng) as control of transfection efficiency. The cell lysates were harvested at 36 h post-transfection and subjected to luciferase assay with Luciferase Assay System (Promega).

### Co-immunoprecipitation assay

Transfected or infected cells were washed twice with cold PBS and lysed in lysis buffer (1% TritonX-100, 150 mM NaCl, 20 mM HEPES pH 7.5, 10% Glycerol, 1 mM EDTA) with protease inhibitor cocktail (Roche) for 20 min, then centrifuged at 12,000 rpm at 4°C for 10 min. The supernatants were incubated with indicated antibody at 4°C for 1 h, then the protein A-Sepharose beads were added and rotated at 4°C for 3 h and washed with lysis buffer. The bound proteins were eluted by boiling with SDS loading buffer for 5 min and subjected to Western blot.

### GST pull-down assay

GST-fused PB1 or PB2 and the corresponding truncated mutants were bound onto glutathione-Sepharose beads (GE Healthcare), then mixed with His-Hsp70 or its mutants in lysis buffer at 4°C for 60 min, the beads were washed with lysis buffer, and the bound proteins were seperated by SDS-PAGE followed by Western blotting with monoclonal anti-His antibody.

### RNA interference

RNA interference was carried out using siRNA purchased from Invitrogen. The siRNA corresponding to the Hsp70 mRNA sequences 5′-UGC ACC UUG GGC UUG UCU CCG UCG U-3′ was used to inhibit endogenous Hsp70 protein expression. Control siRNA sequence was 5′-UGC GUC GUC GAU CGC UUA CUC UCG U-3′. Transfection of siRNA into HeLa cells was performed according to the manufacturer's instructions. In brief, HeLa cells at 50% confluence were transfected with 40 nM siRNA using the Lipofectamine 2000.

### Immunofluorescence

A549 cells were plated on glass coverslips and infected with virus. At indicated time after infection, the cells were fixed with 4% paraformaldehyde for 10 min and permeabilized with 1% TritonX-100 in PBS for 10 min. Then the cells were incubated with rat anti-NP and rabbit anti-Hsp70 antibodies at 37°C for 1 h, washed with 0.5% NP-40 in PBS and incubated with TRITC-conjugated donkey anti-rabbit antibody and FITC-conjugated goat anti-mouse antibody for 1 h, then stained with 2 µg/ml 4′, 6′-diamidino-2-phenylindole dihydrochloride (DAPI). The cells were mounted and observed under confocal microscope (Leica).

### Glycerol density gradient centrifugation

MDCK cells were infected with rWSN at MOI of 10 for 1 h and cultured for 6 h, then the cells were harvested and incubated in hypotonic lysis buffer (10 mM Tris, pH 8.0, 10 mM NaCl, 3 mM MgCl_2_, and 1 mM EGTA with protease inhibitors and RNase Inhibitor) for 10 min, followed by 20 strokes of a Dounce B homogenizer. The lysates were spun down for 5 min at 500 g, and the nuclear pellet was washed three times in wash buffer (hypotonic lysis buffer with 0.1% Nonidet P-40) and then treated with nuclear lysis buffer (20 mM HEPES, pH 8.0, 25% glycerol, 0.42 M NaCl, 1.5 mM MgCl2, and 0.2 mM EDTA with a mixture of protease and RNase inhibitors) for 30 min. The sample was spun at 14,000 g for 30 min, and the supernatant was designated as the nuclear fraction. The nuclear fraction was incubated with 20 µg of His-Hsp70 at 4°C for 1 h or BSA as negative control, and then loaded onto one step glycerol gradient (1 ml of 70%, 0.75 ml of 50%, 0.375 ml of 40% and 1.8 ml of 33% (wt/vol) glycerol). The gradients were centrifuged at 39,000 rpm for 4 h at 4°C in a SW40Ti rotor (Beckman). Fractions (300 µl) were collected from top to bottom.

### Quantitative real-time RT-PCR

Total RNA was extracted from cells by TRIzol Reagent (Invitrogen) according to the instruction. The reverse transcription was carried out using sense-specific oligonucleotides for vRNA (5′ AGC GAA AGC AGG 3′ and 5′ AGC AAA AGC AGG 3′), cRNA (5′ AGT AGA AAC AAG G 3′), and mRNA [oligo(dT)_18_]. A glyceraldehyde-3-phosphate dehydrogenase (GAPDH)-specific primer (5′ GAT GCA GGG ATG ATG TTC 3′) was also included in the RT reaction mixture for vRNA and cRNA. The quantitative real-time PCR was carried out in 20 µl reaction of a mixture containing primers for each of the eight RNA segments [51] or GAPDH (sense primer 5′ TGC ACC ACC AAC TGC TTA G 3′, antisense primer 5′ GAT GCA GGG ATG ATG TTC 3′) with SYBR green DNA dye (TOYOBO). The amount of viral RNA, expressed as threshold cycle (C_T_) values, was normalized by the amount of GAPDH RNA. Primers of semiquantitative RT-PCR for NP gene: sense primer is 5′-CAGATACTGGGCCATAAGGAC-3′ and antisense primer is 5′-TTGTCTCCGAAGAAATAAG-3′

### Mice and virus infection

TAT was designated as YGRKKRRQRRR, 11-amino-acid peptide of human immunodeficiency virus (HIV) TAT protein. TAT-Hsp70 and TAT-GFP [58], [59] were amplified by PCR, inserted into pET28c, expressed in *E. coli* strain BL21 (DE3) pLysS and purified. Specific-pathogen-free four-week-old female BALB/c mice were used in the studies. Experiments were performed in accordance with the institutional guidelines. Mice were divided into three groups (15/each group). On day 0, mice were anesthetized with ether and infected intranasally with 10^4^ PFU of rWSN mixed with 200 µl of PBS (group 1), TAT-GFP (group 2) or TAT-Hsp70 (group 3) (1 µg/µl). From day 1 to day 6, mice were injected intraperitoneally once a day with 200 µl of PBS (group 1), TAT-GFP (group 2) or TAT-Hsp70 (group 3) (1 µg/µl). Three mice from each group were sacrificed on day 3 post-infection, and viral titers in lung were determined by plaque assay. The remaining 12 mice in each group were examined daily for their body weight and survival.
